# Origin of radiation resistance in multi-principal element alloys

**DOI:** 10.1038/s41598-018-34486-5

**Published:** 2018-10-30

**Authors:** Hyeon-Seok Do, Byeong-Joo Lee

**Affiliations:** Department of Materials Science and Engineering, Pohang University of Science and Technology (POSTECH), Pohang, 37673 Republic of Korea

## Abstract

Using molecular dynamics simulations, we characterized the generation and evolution of radiation-induced point defects in the CoCrFeMnNi high-entropy alloy (HEA), to compare it with pure Ni and pure Fe. The generation of primary point defects was investigated by a cascade simulation at 773 K and the evolution of point defect clusters by a defect evolution simulation using 1 at% defect-containing samples. The numbers of residual defects after cascade and surviving defects after evolution in the CoCrFeMnNi HEA are smaller than those in pure Ni and pure Fe. The defect clusters appearing in the CoCrFeMnNi HEA after the defect evolution are unstable because of the alloy complexity. The origin of the slower radiation damage accumulation and the higher radiation damage tolerance in the CoCrFeMnNi HEA is discussed.

## Introduction

Recently, high-entropy alloys (HEAs) have been drawing much attention as new structural materials due to their structural stability and excellent mechanical properties^1^. Unlike the conventional metallic alloys composed of one or two principal elements, HEAs consist of five or more major elements which have an almost equiatomic ratio^2^. Due to the alloy complexity, HEAs have been reported to have a wide range of outstanding material properties compared to the conventional alloys^3–6^. In particular, most HEAs exhibit high radiation damage tolerance as reported by experimental studies^7–10^, so HEAs also attract attention as new nuclear materials. However, to be used as actual nuclear materials, composition or element change in widely known HEAs, such as the CoCrFeMnNi HEA, may be needed. For example, cobalt should be replaced by another element due to safety and cost problems^11^. Therefore, for efficient alloy design, it is necessary to understand how the characteristics of HEA affect the radiation damage tolerance.

Radiation damage is caused by radiation-induced defects and their evolution such as voids and dislocation loops. Therefore, the primary radiation damage process is an atomic-level materials phenomenon. Simulation analyses have been carried out in many experimental studies^7,12–14^ because it is difficult to accurately analyze the atomic-level defect generation and evolution mechanism through experimental techniques alone. As a result, the difference in the defect migration direction between the HEA and conventional alloys, the reduction of the migration energy difference between different types of defects^7^, the decrease of defect and dislocation mobility^12,15^, and the change in the energy dissipation process^13^ due to the alloy complexity have been mentioned as the reasons for the high radiation damage tolerance of HEAs. However, these results are mainly inferred from the phenomena observed in binary or ternary Ni-containing FCC single-phase concentrated solid solution alloys (SP-CSAs). Further, it is unclear how these reasons affect the generation of the radiation-induced defect and the formation of defect clusters.

In this study, an atomistic simulation on the quinary equiatomic CoCrFeMnNi HEA was performed to clarify the reasons for the high radiation damage tolerance of HEAs. We investigated the effect of the alloy complexity of the HEA on radiation-induced defect generation and evolution using a displacement cascade simulation and a defect evolution simulation, and confirmed its effect on radiation damage tolerance.

## Results

### Primary defect generation

**Figure 1:**
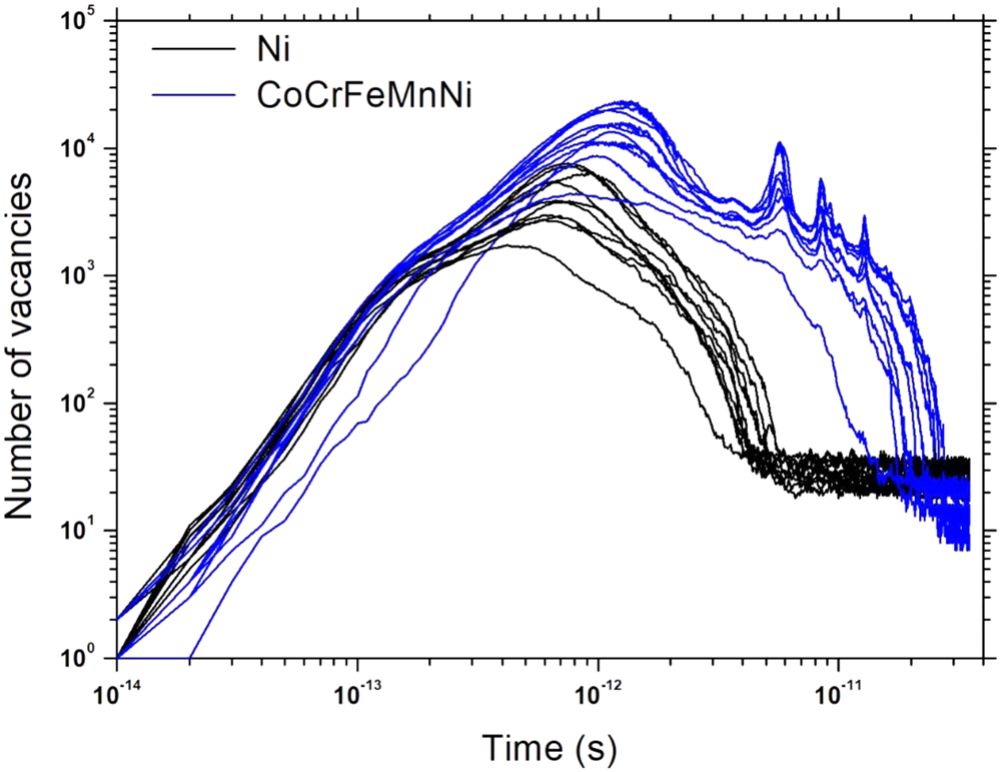
The number of vacancies generated during 10 cascade simulations with 10 keV PKA energy at 773 K, for pure Ni (black curves) and CoCrFeMnNi HEA (blue curves).

**Figure 2:**
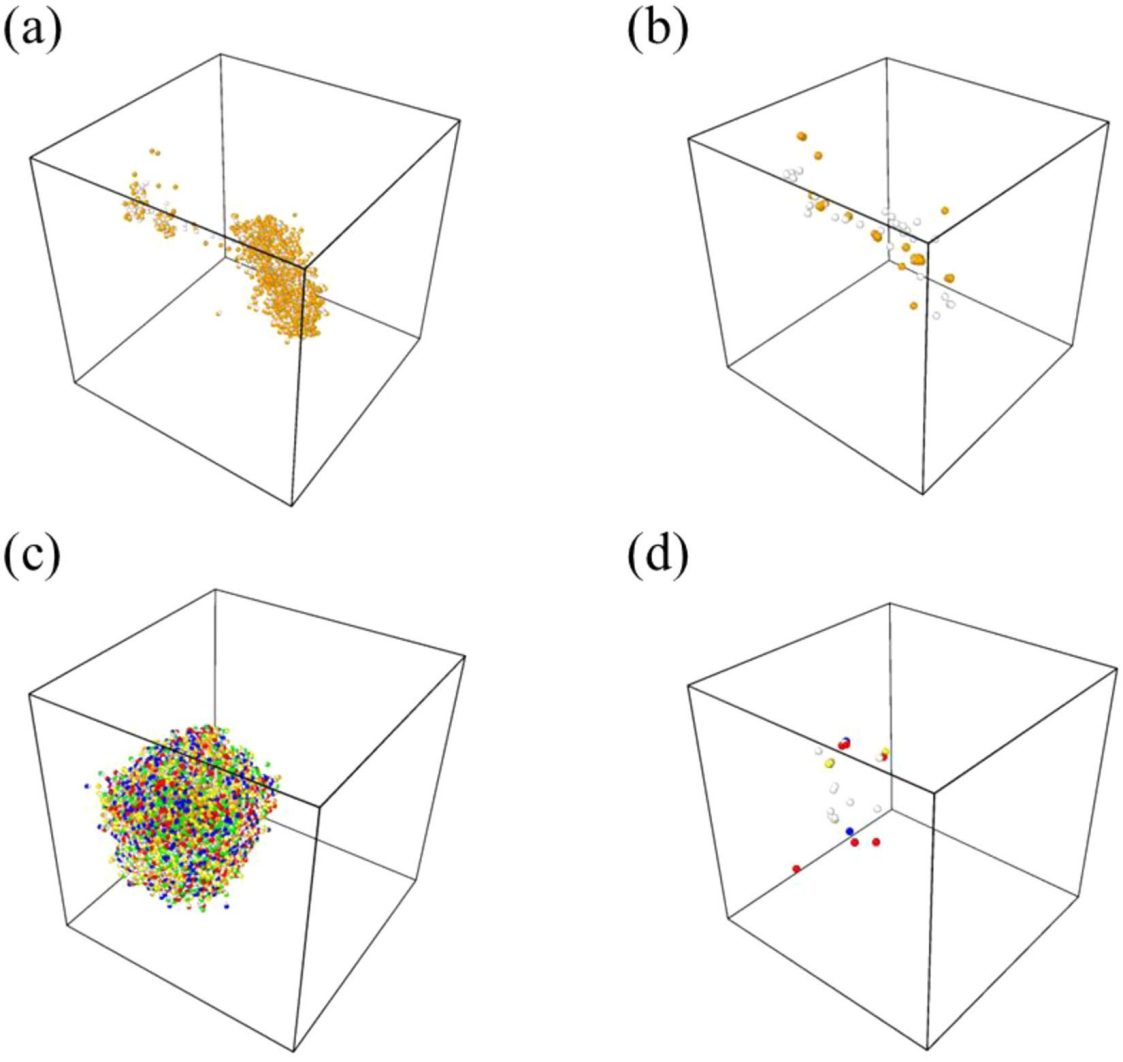
A snapshot of defect distribution (**a**,**c**) at the defect generation peak time (1 ps) and (**b**,**d**) at the end of the cascade simulation (35 ps) in (**a**,**b**) pure Ni and (**c**,**d**) CoCrFeMnNi HEA. The red, orange, yellow, green, blue and white circles represent Co, Ni, Cr, Fe, Mn interstitials and vacancies, respectively.

Figure 1 presents the number of primary defects generated by a recoil after irradiation, and Table 1 summarizes the number of residual defects after 35 ps. The CoCrFeMnNi HEA has more peak defects than pure Ni at the defect generation peak time, but after recombination of defects, there were fewer residual defects. Although secondary peaks appear due to the elastic wave traversing the cell triggering formation of many defects, it has been reported that secondary peaks have no effect on final damage of cascade simulation^16^. According to a previous study^17^, the number of peak defects in pure BCC Fe under the same PKA energy recoil is one third of that of the CoCrFeMnNi HEA at similar temperatures. However, as presented in Table 1, the CoCrFeMnNi HEA has fewer residual defects than even the pure Fe. Figure 2 presents the distribution of defects at around the peak time of defect generation (1 ps) and the distribution of residual defects after the end of the cascade simulation (35 ps) for pure Ni and the CoCrFeMnNi HEA. In the pure Ni, as the cascade propagates, subcascades are formed and the defects are generated along the PKA recoil direction, resulting in a widespread distribution of residual defects. Pure Fe is also known to exhibit primary defect generation characteristics similar to the pure Ni under the same PKA energy recoil^18^. The CoCrFeMnNi HEA, on the other hand, rarely form the subcascade during cascade propagation and has no directionality of defect generation along the direction of the PKA recoil. Therefore, the residual defects in the CoCrFeMnNi HEA are confined to a relatively limited space.

**Table 1:** The number of surviving Frenkel pairs in FCC CoCrFeMnNi HEA and pure Ni, averaged from 10 cascade simulations with 10 keV PKA energy at 773 K. The results for pure BCC Fe are from previous studies with the same PKA energy at temperatures similar to 773 K (600, 673, and 900 K).

Alloys	Temperature(K)	Number of Frenkel pairs (stdev)
CoCrFeMnNi HEA	773	13.5 (4.6)
Pure FCC Ni	773	23.9 (4.3)
Pure BCC Fe	600	17.4^17^, 25^30^
	673	22.9^29^
	900	27.3^18^

### Defect evolution

**Figure 3:**
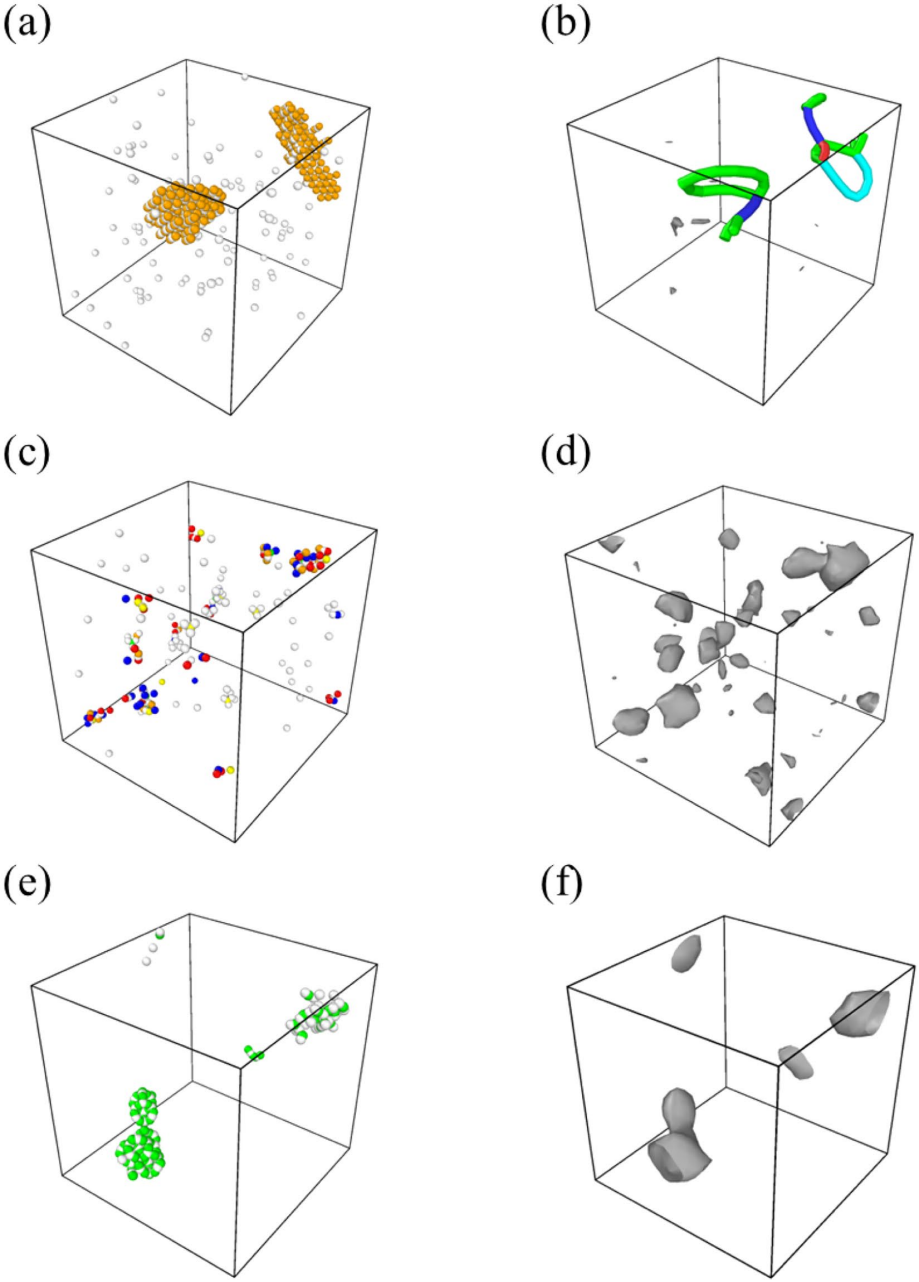
(**a**,**c**,**e**) The defect distribution and (**b**,**d**,**f**) its dislocation extraction analysis (DXA) after 5 ns defect evolution in (**a**,**b**) pure Ni, (**c**,**d**) CoCrFeMnNi HEA and (**e**,**f**) pure Fe. In DXA, blue, green and turquoise lines represent perfect dislocations, partial dislocations and the Frank dislocation loop, respectively. Red lines are dislocations which cannot be recognized by DXA and dark grey meshes represent defect meshes.

To examine how the defects evolve in each material system, MD simulations at 773 K were performed on samples involving a randomly distributed, large number (1%) of residual Frenkel pairs. Table 2 summarizes the number of remaining defects and Fig. 3 presents the distribution of the remaining defects in FCC CoCrFeMnNi HEA, pure Ni and pure BCC Fe, after 5 ns defect evolution simulation. As presented in Table 2, the fraction of the remaining defects is small in the CoCrFeMnNi HEA compared to pure systems, which means that the defects in the CoCrFeMnNi HEA have a high probability of recombination during the evolution. Since the number of Frenkel pairs in large clusters cannot be accurately counted by the displaced-atom method we used in this study (see method section), we also performed another defect analysis for pure Ni and the HEA using a Wigner-Seitz cell method (Supplementary Table S1). The results from the Wigner-Seitz analysis show a similar trend with those from the displaced-atom method in Table 2.

**Table 2:** The number and fraction of the remaining displaced atoms after a 5 ns defect evolution of FCC CoCrFeMnNi HEA, pure Ni and pure BCC Fe samples containing randomly distributed initial defects of 1%, averaged from four simulations at 773 K.

Alloy	Number of remaining displaced atoms (stdev)	Fraction of remaining displaced atoms (stdev)
CoCrFeMnNi HEA	85.3 (12.1)	0.27 (0.04)
Pure FCC Ni	167.3 (56.1)	0.52 (0.18)
Pure BCC Fe	87 (1.4)	0.54 (0.01)

As presented in Fig. 3(a,b), in pure Ni, the vacancies are present in the form of single defects without deviating from their initial position, while self-interstitial atoms gather and form stable and rarely mobile SIA clusters with a dislocation loop, as reported from previous experiment and simulation^7,19^. In the case of pure BCC Fe, as presented in Fig. 3(e,f), SIAs form immobile defect clusters and the remaining defects are gathered. The SIA clusters in pure BCC Fe has been reported to be stable at about 773K^20,21^. In the CoCrFeMnNi HEA, defects exist in the form of single defects as well as defect clusters. However, the defects move mainly as individual single defect instead of clusters during defect evolution, unlike the defects in pure Ni that move in a form of interstitial clusters containing dislocations. In addition, as presented in Fig. 3(d), the defect clusters in the CoCrFeMnNi HEA are represented by defect meshes because the defect clusters contain no dislocation. These differences indicate that the defect clusters in the CoCrFeMnNi HEA are different from those in pure FCC Ni and that the motion of defects in the CoCrFeMnNi HEA is not mainly dislocation movement.

## Discussion

From the smaller number of residual defects in the CoCrFeMnNi HEA under the same recoil conditions, one can expect that the radiation damage accumulation in the HEA would be lower than in pure Ni and pure Fe. The distribution of generated defects in a relatively confined space would increase the possibility of recombination, so the smaller number of residual defects in the CoCrFeMnNi HEA may be attributed to the limited distribution of defects. Therefore, it is necessary to explain the causes of the difference in the defect distribution. The difference in the defect distribution in the CoCrFeMnNi HEA, pure Ni and pure Fe can be explained by the difference in the vacancy and self-interstitial atom (SIA) migration energy and in the threshold displacement energy of each material. The calculated migration energy of the vacancy and SIA for each material are presented in Table 3, and the calculated threshold displacement energies are presented in Table 4 in comparison with available experimental information. The migration energy of the vacancy and SIA were obtained through a mean-square displacement (MSD) calculation at finite temperatures. The migration energy calculated through this method considers various local atomic configurations in overall. Therefore, the fluctuation should be much smaller than in the calculation of individual migration energy. It should be noted that the migration energy of the SIA in the CoCrFeMnNi HEA was much higher than those in pure metals. The vacancy migration energy in the HEA was similar to the SIA migration energy, while there was a big difference between those quantities in pure Ni. The migration energy of the two defects in pure Fe is generally smaller than those in other materials. Ni is the element with the highest vacancy migration energy among the five components in the CoCrFeMnNi HEA. Therefore, it is natural for the equiatomic five-component alloy to show an intermediate average value, smaller than that of pure Ni. The large SIA migration energy in the CoCrFeMnNi HEA was an unexpected result. We believe that the large SIA migration energy comes from the variety in the local environment of each interstitial site. Some neighboring distribution of atoms may provide a stable environment (local distortion and relaxation) to a SIA and may increase the energy barrier for the migration of the SIA. The degree of disorder associated with defects in the HEA evaluated by calculating the fluctuations in the formation energy of vacancy and Frenkel pairs. The details of the analysis are provided as supplementary information of this manuscript. It should also be emphasized here that the threshold displacement energy that is calculated at 0 K can be uniquely defined in pure elements, but not in the multi-component HEA due to the variety in the environment of individual lattice points. Therefore, we obtained the threshold displacement energy for the HEA from several calculations for several different samples and took the average. In Table 4, the minimum values of the threshold displacement energy in individual directions are presented in addition to the average values, for the CoCrFeMnNi HEA.

**Table 3:** Migration energy barrier E_m_ of single vacancy and self-interstitial atoms in FCC CoCrFeMnNi HEA, pure FCC Ni and pure BCC Fe. For the CoCrFeMnNi HEA, the result is an average from 5 different random solid solution samples with different configurations.

Alloy	E_m_(eV)	
	Single vacancy	Self-interstitial atom
CoCrFeMnNi HEA	0.79 (0.12)	0.61 (0.08)
Pure FCC Ni	1.46	0.26
Pure BCC Fe	0.27	0.11

**Table 4:** Calculated and experimental threshold displacement energy of FCC CoCrFeMnNi HEA, pure Ni and pure Fe for <100>, <110> and <111> directions. For the CoCrFeMnNi HEA, the result is an average from 20 different random solid solution samples with different configurations. The unit of the threshold displacement energy is eV.

Alloy		Crystallographic directions		
		<100>	<110>	<111>
CoCrFeMnNi HEA	Present work, Average (stdev)	26.2 (5.9)	29.1 (5.7)	64.3 (15.8)
	Present work, Minimum value	16	19	38
Pure FCC Ni	Present work	34	43	52
	Experiment^34^	38	21	>60
Pure BCC Fe	2NN MEAM^*^^31^	22	32	29
	Experiment^35^	17	>30	20

^*^Previous study using 2NN MEAM potential.

As presented in Tables 3 and 4, the minimum value of the threshold displacement energy in the CoCrFeMnNi HEA is relatively smaller than that of pure Ni and pure Fe, while the SIA migration energy is higher. During the displacement cascade simulation, the cascade propagation mainly leads to a SIA migration as the PKA energy is much larger than conventional threshold displacement energy. Therefore, the cascade propagation in pure Ni with a small SIA migration energy would be relatively long and last for a long time because of the small PKA energy loss due to the SIA movement. On the other hand, in the CoCrFeMnNi HEA which has a large SIA migration energy, the cascade propagation would not be sustained for a long distance due to the large PKA energy loss. Because of the lattice distortion that comes from the diversity of constituent elements, the cascade would propagate in various directions, not only along the initial PKA direction. In addition, the relatively small threshold displacement energy in the CoCrFeMnNi HEA would contribute to generating many defects in various directions, resulting in preventing the formation of subcascades and increasing the number of defects. Because of the low SIA migration energy and relatively low lattice distortion, it is also expected that the generation characteristic of defect in pure Fe would be similar to that in pure Ni.

We concluded that the fewer residual defects in the CoCrFeMnNi HEA than in pure Ni and pure Fe originates from the relatively confined defect distribution caused by the high SIA migration energy and the lattice distortion. The directional cascade propagation in pure Ni and less directional cascade in Ni-Fe and Ni-Co binary alloys have been observed by previous experimental studies^22,23^. Our results support such a change in the cascade propagation behavior between pure elements and alloys, and confirm that the non-directional cascade propagation also occurs in the multi-component CoCrFeMnNi HEA. This result suggests that not only the CoCrFeMnNi HEA but also various multi-component HEAs can generally be expected to have a similar defect generation characteristic, few residual defects, fewer than in BCC Fe.

The displacement cascade simulation which analyzes one recoil may not be sufficient to estimate the radiation damage tolerance of materials. Because a long-term irradiation would induce an internal microstructure change of the material, it is necessary to grasp the cumulative effect of defects generated by the long-term irradiation, that is, the evolution of residual defects. Since the recombination of the residual defects during the defect evolution occurs more frequently in the CoCrFeMnNi HEA compared to the pure FCC Ni or pure BCC Fe, it can be expected that the initial generation and growth of radiation-induced defect clusters would occur more slowly in the CoCrFeMnNi HEA. It should be noted here that the retardation of the growth of radiation-induced defect clusters in the CoCrFeMnNi HEA has been experimentally reported^7,19^ as an improvement of radiation damage tolerance due to the inhibition of radiation damage accumulation. To explain the cause of the high radiation damage tolerance in the CoCrFeMnNi HEA, we thought it was necessary to clarify the origin of the differences in the recombination rate of residual defects and the defect distribution during the defect evolution. For this, we focused on the stability of defect clusters and the migration energy of defects in each system.

**Figure 4:**
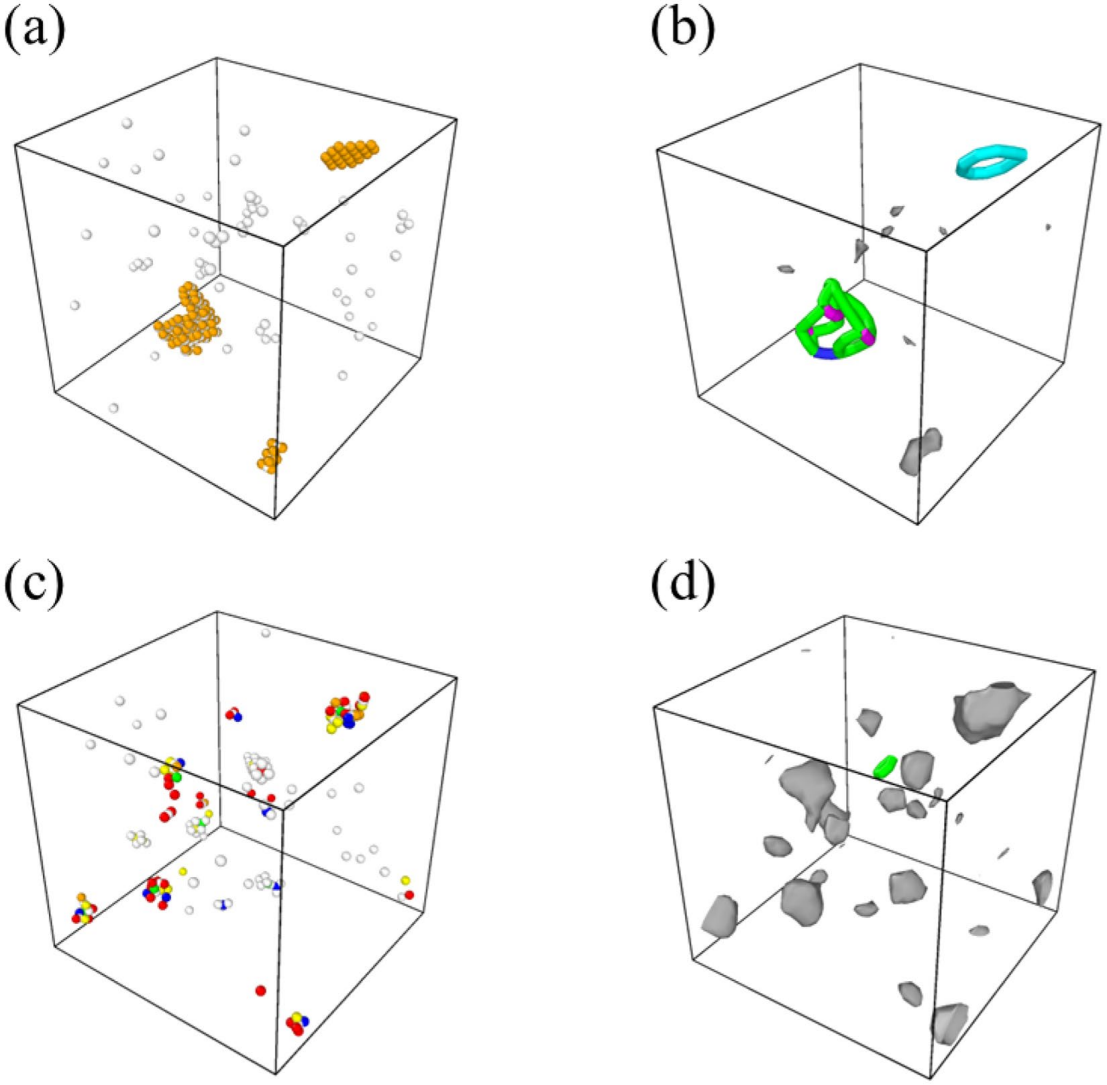
(**a**,**c**) The defect distribution and (**b**,**d**) its dislocation extraction analysis (DXA) (**a**,**b**) after a 5 ns further defect evolution of the sample in which all atoms in the final sample after CoCrFeMnNi HEA 5 ns defect evolution are changed to Ni and (**c**,**d**) after a 15 ns further defect evolution of the final sample after CoCrFeMnNi HEA 5 ns defect evolution without change in the atom type.

First, because the shape of defect clusters in the CoCrFeMnNi HEA was different from that of the stable defect clusters in the conventional FCC systems, we performed two additional defect evolution simulations to confirm the stability of the defect clusters in the CoCrFeMnNi HEA: one changing all the atoms into Ni atoms after the 5 ns evolution simulation on the CoCrFeMnNi HEA sample and then continuing a further defect evolution simulation, and the other simply continuing a further defect evolution simulation without changing the atom types. The defect distribution after the additional defect evolution simulation is presented in Fig. 4. As presented in Fig. 4(b), defect clusters in the sample in which all atoms have been changed to Ni are mostly transformed into dislocation-containing defect clusters that are stable in the conventional FCC system and lose their original shape. However, as presented in Fig. 4(d), the defect clusters in the unchanged sample retain the original shape and are rarely transformed into stable dislocation-containing defect clusters even after the 15 ns additional defect evolution simulation. These results indicate that the defect clusters in the CoCrFeMnNi HEA are less stable than those in pure FCC system, quite probably because of the lattice distortion due to the alloy complexity. The insufficient stability of defect clusters in the CoCrFeMnNi HEA would yield the existence of the defects as single defects or small defect clusters that can migrate and recombine easily rather than stable and immobile clusters.

Second, concerning the differences in the distribution of clusters, one needs to pay attention to the migration energy differences of defects among materials. As presented in Table 3 the vacancy migration energy in pure Ni is much higher than in other materials while the SIA migration energy is low, and this must be the reason for the existence of the single vacancies and stable SIA clusters in pure Ni. In this case, the probability of recombination of the defects would be low and the fraction of remaining defects remains high, as presented in Table 2. The migration energy of both defects in pure Fe is relatively low, so the probability of recombination of defects is expected to be relatively high. However, contrary to the expectation, the fraction of the remaining defects in pure Fe is relatively high. This is because the SIAs form stable clusters and the remaining defects are also gathered, not recombined. Although the migration energy of both defects in the CoCrFeMnNi HEA is relatively high compared to that in pure Fe, the defects exist in the form of single defects or small clusters which have relatively high mobility. The instability of SIA clusters and the rather high mobility of the defects would delay the growth of defect clusters and enhance the recombination of the defects. Overall, in addition to the relatively limited spatial distributions of defects already discussed, we propose the formation of relatively small clusters and the intermediate migration energy barrier of both defects as the reason for the relatively high recombination rate and the low fraction of the remaining defects in the HEAs. Since the instability of SIA clusters and the limited spatial distribution of defects in the HEA originate from the lattice distortion due to the chemical complexity, similar improvement in radiation resistance can be generally expected in multi-principal element alloys.

## Conclusion

To summarize, we have demonstrated that the CoCrFeMnNi HEA has a lower radiation damage accumulation than pure FCC Ni and pure BCC Fe. In the CoCrFeMnNi HEA, defects are generated in a relatively limited space during the radiation-induced defect generation because of the high SIA migration energy and the absence of a subcascade, which results in a high proportion of recombination and a small number of residual defects. In addition, the instability of defect clusters due to the lattice distortion further increases the recombination rate of residual defects and delays the growth of the defect cluster. The retardation of radiation damage accumulation yields the high radiation damage tolerance in the CoCrFeMnNi HEA. Since the origin of the retardation of radiation damage accumulation in the CoCrFeMnNi HEA is the lattice distortion due to the alloy complexity, high radiation damage tolerance can also be expected from a wide range of multi-principal element alloys.

## Method

The influence of alloy complexity on the radiation damage tolerance in the HEA was investigated by performing a comparative molecular dynamics (MD) simulation using the LAMMPS^24^ code on the CoCrFeMnNi HEA, pure FCC Ni and pure BCC Fe. We utilized two MD simulation methods: a displacement cascade simulation and a defect evolution simulation. The displacement cascade simulation was conducted to analyze the characteristics of the primary defect generation in the initial irradiation environment. In the displacement cascade simulation, a recoil of primary knock-on atom (PKA) creates residual defects deviating from their original lattice sites permanently. It is important to analyze the cumulative effect of residual defects because the generation of many residual defects due to long-term irradiations and the interaction among them cause changes in the microstructure of the nuclear structural materials. Therefore, various atomistic simulation techniques have been conducted to estimate the residual defects evolution. For example, the evolution of residual defects was analyzed using hybrid MC/MD simulations through a multiple cascade^25^ and a kinetic Monte-Carlo (KMC) or MD simulation on a sample with point defects^15,26^. In this study, we chose the defect evolution simulation method which performs the MD simulation at a specific temperature on a sample containing randomly-distributed point defects in order to most efficiently analyze the interaction among residual defects given as point defects at a specific temperature.

An interatomic potential reported by Choi *et al*.^27^ based on the 2NN MEAM interatomic potential formalism^28^ was used to represent atomic interactions in the CoCrFeMnNi HEA as well as pure Ni and pure Fe. The potential was the only potential that described the atomic interaction in the CoCrFeMnNi HEA reproducing atomic-level phenomena in the CoCrFeMnNi HEA well^27^. However, since short-range repulsion for atomic collision is important in the radiation damage simulation, we checked the performance of the potentials for individual five elements before carrying out cascade simulations. In pure Co and pure Mn, the short-range repulsion was not sufficient and the primary knock-on atom (PKA) passes through the neighboring lattice site without colliding with the atom there as if there were no atom on the lattice site. Therefore, the d parameters in unary Co and Mn systems were minimally adjusted from zero to 0.03 and 0.02, respectively, so that the atomic collision occurs in typical test directions, <100>, <110> and <111>. It was also confirmed that the change of the d parameter had little effect on the fitted physical properties in each unary system. All simulations conducted in this study were performed with a radial cutoff distance 4.5 Å for FCC structures and 3.6 Å for pure BCC Fe; these sizes are larger than the second nearest-neighbor distance. We believe the artifact originates from the incompleteness of the interatomic potential would be small because the potentials for pure elements and the CoCrFeMnNi HEA are based on the same formalism and the description for pure Ni and Fe in the alloy is exactly the same as those for individual elements.

The displacement cascade simulations were performed at 773 K for the CoCrFeMnNi HEA and pure Ni samples using a 50 × 50 × 50 FCC unit cell system containing 500,000 atoms. Since most of the HEAs are prepared by a heat treatment at or above 800°C and by quenching to avoid probable formation of intermetallic compounds at low temperatures, the atomic arrangement of the HEAs would correspond to the arrangement at 800°C or higher. Therefore, for the CoCrFeMnNi HEA, we used a random FCC solid solution equilibrated at the cascade simulation temperature. The sample size was sufficiently large so that the propagation of the cascade did not overlap. The PKA energy was 10 keV, and <135> and <235> were selected as the PKA directions. The time step in the simulations was adjusted so that each atom did not move more than 0.02 Å per one time step, and each recoil lasted 35 ps. We did not perform the displacement cascade simulation for the pure BCC Fe because the analysis of the displacement cascade simulation for the pure BCC Fe has already been discussed in many previous studies^17,18,29,30^. We mainly compared the analysis of several previous studies for the pure BCC Fe with our results for FCC CoCrFeMnNi HEA and pure Ni.

To analyze the evolution of surviving defects from displacement cascades, the defect evolution simulations were also performed on FCC CoCrFeMnNi HEA, pure Ni and pure BCC Fe using a 20 × 20 × 20 unit cell. 1 at% of Frenkel pairs were randomly distributed in each sample to better model the situation in which a large number of point defects are generated by multiple irradiations. The time step in the simulations was 1 fs and each simulation lasted 5 ns at the same temperature as the displacement cascade simulation.

We used a displaced-atom analysis to detect point defects and defect clusters as described in the literature^31^. For each lattice site if there was no atom within 0.3a, the site was determined to be a vacancy site, and an atom that was more than 0.3a away from the nearest lattice site was considered as an interstitial atom, where “a” represents the lattice parameter. A dislocation extraction algorithm (DXA)^32^ in the open source application OVITO^33^ was also used to visualize dislocation lines in defect clusters and distinguish the defect cluster’s shape in the same crystal structure.

## Electronic supplementary material

Supplementary Information
