# Supplementary material for: Origin of radiation resistance in multi-principal element alloys
Source: Sci Rep. 2018 Oct 30;8:16015. doi: 10.1038/s41598-018-34486-5 (PMC6207767; doi:10.1038/s41598-018-34486-5)
Supplement: Supplementary file 1 — Supplementary Information [file 41598_2018_34486_MOESM1_ESM.pdf]

Supplementary Information

Origin of radiation resistance in multi-principal element alloys

Hyeon-Seok Do[1] and Byeong-Joo Lee[1]*

[1]Department of Material Science and Engineering,

Pohang University of Science and Technology (POSTECH), Pohang 37673, Republic of Korea

S1. Evaluation of the degree of disorder associated with the defects in CoCrFeMnNi HEA.

As a means to estimate the degree of disorder associated with the defects, we calculated the fluctuations in the formation energy of vacancies and Frenkel pairs in CoCrFeMnNi HEA samples.

In the case of vacancy formation energy, since the exact calculation is difficult when the composition is changed, we randomly selected each atom of the 5 constituent elements, removed them without changing the overall composition and calculated the formation energy of 5 vacancies. Supplementary Fig. S1(a) shows the fluctuation in the formation energy of 5 vacancies (average value for one vacancy) for 20 different configurations. The vacancy formation energy calculated by this method is 1.56 eV with a standard deviation of 0.07 eV. Supplementary Fig. S1(b) shows the fluctuation in the formation energy of 40 different, arbitrarily created Frenkel pairs. The average is 4.45 eV and the standard deviation is 0.47 eV. The fluctuation in the formation energy of the individual Frenkel pairs is significant (the maximum fluctuation, 2eV, is almost half of the average value, 4.45eV). The fluctuation in the vacancy formation energy is smaller because the values are average values for the 5 vacancies, not those for individual vacancies.

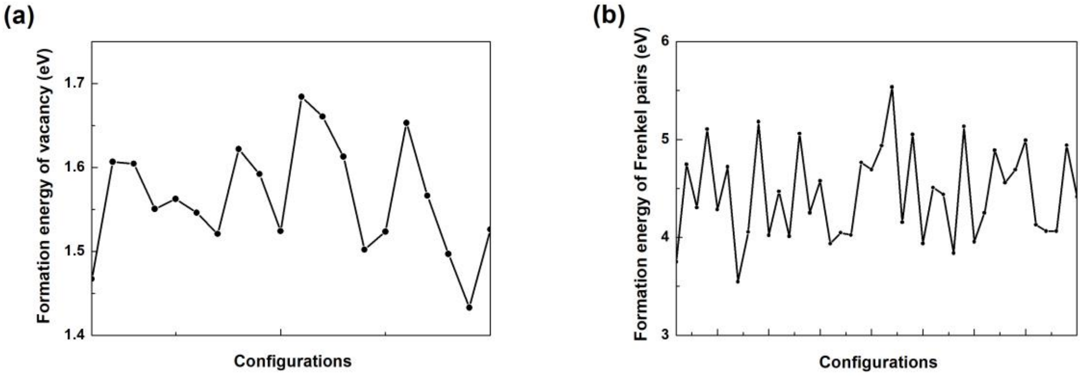

Supplementary Figure S1. The calculated formation energy of (a) vacancy (average of five vacancies from each element) and (b) Frenkel pairs in CoCrFeMnNi HEA, for randomly selected 20 and 40 different configurations, respectively.

S2. Remaining Frenkel pairs analyzed by Wigner-Seitz method after a 5ns defect evolution of CoCrFeMnNi HEA and pure Ni.

Understanding that the number of Frenkel pairs in large clusters cannot be accurately counted by the displaced atom method, we also performed the defect analysis for pure Ni and the HEA using a Wigner-Seitz cell method. The number and fraction of defects after the 5 ns defect evolution (as in Table 2) analyzed by the W-S method are presented in Supplement Table S1.

Alloy	Number of remaining Frenkel pairs (stdev)	Fraction of remaining Frenkel pairs (stdev)
CoCrFeMnNi HEA	60.3 (7.4)	0.19 (0.02)
Pure FCC Ni	91.8 (11.6)	0.29 (0.04)

Supplementary Table S1. The number and fraction of the remaining Frenkel pairs analyzed by Wigner-Seitz method after a 5ns defect evolution of FCC CoCrFeMnNi HEA and pure Ni containing randomly distributed initial defects of 1%, averaged from four simulations at 773K.
